# The Islamic Perspectives of Gender-Related Issues in the Management of Patients With Disorders of Sex Development

**DOI:** 10.1007/s10508-016-0754-y

**Published:** 2016-04-21

**Authors:** Ani Amelia Zainuddin, Zaleha Abdullah Mahdy

**Affiliations:** Department of Obstetrics & Gynaecology, Faculty of Medicine, National University of Malaysia, Jalan Yaakob Latif, Bandar Tun Razak, 56000 Cheras, Kuala Lumpur Malaysia

**Keywords:** Disorders of sex development, Islam, Gender dysphoria, Fatwa, Congenital adrenal hyperplasia, Androgen insensitivity syndrome

## Abstract

In Islam, the person with somatic sex ambiguity due to a disorder of sex development (DSD), such as 46,XX congenital adrenal hyperplasia or 46,XY androgen insensitivity, is recognized as khunsa. Two types of khunsa are distinguished: wadhih (discernible) and musykil (intractable). A recent fatwa (religious edict) in Malaysia decreed that it is permissible for male-assigned patients from these two groups to have gender reassignment surgery to female following diagnosis; however, the religious authority has yet to rule on the reassignment from female to male, if requested. The different schools of law in Islam agree on some aspects of gender-related issues like the position of khunsa in prayer congregations, but differ in their opinions on others such as property inheritance and bathing rituals. For purposes of illustration, this article includes three case reports on Muslim patients with DSD in Malaysia, focusing on issues of gender assignment: (1) a patient with 46,XX CAH, assigned as female, requesting reassignment to male; (2) a patient with 46,XX CAH, assigned female, and gender dysphoric, but undecided on the gender to be; and (3) a patient with 46,XY complete gonadal dysgenesis, raised female due to her phenotype at birth, diagnosed late, at age 18 years, and content to remain female. Gender-related issues from the perspective of Islamic jurisprudence are highlighted and discussed. To ensure holistic care, health-service providers involved in the care of Muslim patients with DSDs need to be aware of the Islamic perspectives on gender-related issues and involve expert religious authorities.

## Introduction

Management of patients with disorders of sex development (DSD) is undoubtedly challenging. Key tasks that, over the past few decades, have caused much controversy among patients, their families, and clinicians involved in their care include (1) gender assignment, (2) gender-affirming surgery, and (3) disclosure of information. In this context, due consideration must be given to culture and religious factors.

Culture plays an important role in the gender determination of patients with atypical somatic sex development (Kuhnle & Krahl, 2002; Meyer-Bahlburg, 1998). Cultural influences may contribute to patients with DSD and their families’ acceptance or rejection of their assigned gender, to the psychosexual development of the patient, and medical management. There are reports from several countries such as Saudi Arabia (Taha, 1994), Turkey (Özbey, Darendeliler, Kayserili, Korkmazlar, & Salman, 2004), and Egypt (Dessouky, 2001) that indicate increased rates of assignment to the male gender regardless of karyotype, gonadal makeup, and fertility potential, because the male gender has a dominant role in society and is thus the preferred sex. In India and Pakistan, DSD children are more likely to be raised as males simply in order to ensure a better future for these children when they grow up (Warne & Raza, 2008). Even if they are infertile as males, they are more likely than infertile females to achieve economic independence.

Nevertheless, not all Muslim countries have a preference for the male sex. The majority of patients with DSD in Malaysia are Muslims as it is a predominantly Muslim country. Kuhnle and Krahl (2002) reported that, in their experience of working in the largest children’s hospital in Malaysia, “it was never difficult to convince a Muslim family to assign a severely virilized girl or an undervirilized boy to the female gender. This was not the case for Chinese and Indian families, who on several occasions took off with their ambiguously born child when female sex assignment (or reassignment) was suggested.” This was because in Malaysia, Malay Muslim women are entitled to inherit and control their own money, and with divorce, or when widowed, a woman’s fortune remains under her control, enabling her to be independent. It was their impression that within the ethnic Chinese and Indian communities of Malaysia, much more prestige was associated with the male role and greater importance to the male offspring, similar to the situation in India and China.

The aim of this article is to report three cases of gender assignment in Malaysia, a fast-developing conservative and moderate Muslim country, with the intention to highlight and increase awareness of the Islamic perspectives of gender-related issues to be considered in the counseling and management of Muslim patients with DSDs. These cases will be briefly introduced, followed by a presentation of the Islamic perspectives with regard to individuals with a DSD.

## Case Reports

### Case 1: Reassignment From Female to Male of a Patient With 46,XX CAH

AA is an 18-year-old Muslim patient with the salt-wasting variant of 46,XX CAH, who had been diagnosed at 1 month of age and assigned female. She underwent feminizing surgery (clitoroplasty) at 5 years of age. Due to limited parental understanding of the condition, medication adherence was extremely poor. Presumably secondary to the resulting androgen exposure and related virilization, AA’s gender role behavior was masculinized since the age of 7 years, and the patient was adamant about being a “boy.” However, the parents insisted that AA wear female clothes and behave as a girl, which caused much family conflict. AA never attained menarche and refused to see a gynecologist to whom she was referred to settle the menstrual problem. Her gender issues included the refusal to pray, as she refused to wear the female Muslim praying attire and demanded to sit with the men at religious functions, and the desire to marry a girl and be a good husband.

Once she became a legal adult at the age of 18 years, she formally requested to change her gender to male. This resulted in a multidisciplinary meeting with the patient, her family, and the doctors involved in her care. Her case was then presented to the National Shariah Council of Malaysia. The religious council was very sympathetic to the patient’s plight, and advised her to pray as a man for now. She was also advised to have regular sessions with a psychiatrist to ensure that she is fully prepared psychologically for the gender change.

### Case 2: Gender Dysphoria in a Patient With 46,XX CAH

BB is a 22-year-old Muslim patient who had been diagnosed at birth as 46,XX CAH with the salt-wasting variant, assigned to the female gender, and had undergone feminizing genitoplasty at 2 years of age. She also had a history of poor medication adherence. Unfortunately, she did not complete secondary school due to psychological problems that stemmed from her unhappiness, because she identified very strongly with the male gender since age 13 years. She was seen by a psychologist. She has always been sexually attracted to females but denies having had any sexual activity. She initially requested formal gender reassignment to male but after further discussion with her parents and acknowledging the process she needs to undergo to legally change her gender status, she was hesitant and asked for more time to think about it. Hence, at present, her gender status is as yet undetermined.

### Case 3: A Patient With 46,XY Complete Gonadal Dysgenesis Raised Female

CC is an 18-year-old Muslim woman who presented with primary amenorrhea and pubertal delay was diagnosed as 46,XY CGD, and underwent gonadectomy. As she had presented with a female phenotype at birth, she had been raised as a female and developed a consistently female gender identity. She has responded well to hormonal replacement therapy in terms of breast and uterine development and is a content young woman, doing well in university. The only issue she and her family needed to come to terms with is her infertility and finding a husband who can accept her as she is.

## Islamic Perspectives on Gender

In the Holy Quran, Allah the Almighty decrees that all human beings, whether male or female, are descended from Adam and Eve (Surah An-Nisa 4:1). Men and women in Islam have different roles, responsibilities, and accountabilities, as they differ in anatomy, physiology, and psychology. Islamic rites are the same for men and women except that women, while menstruating, are not permitted to perform their prayers (solah), fast, recite the Quran, or perform the Tawaf during the Hajj pilgrimage. (Once in their lifetime, if they can afford the journey, Muslim men and women go to Saudi Arabia to perform the hajj pilgrimage in Mecca and Medina. One of the compulsory rituals involved in this pilgrimage is the Tawaf, where the Muslims move counter-clockwise around the Kaabah seven times.) The exclusion from these activities are meant to give women a rest, as many find menstruation discomforting, and not because the menstrual blood is considered to be dirty.

Islam places great significance on the structure of the family as the basic unit of society, in which men and women have important roles to ensure the maintenance of the family. Islam not only sanctifies the life of an individual, but also tries to sacralize the social structures themselves by bestowing a religious significance upon all social institutions and functions and by constituting within society relationships and rapports in order to integrate all these different elements into a single people or ummah (Nasr, 1993).

### Islamic Definitions

Classical Islamic law, in terms of assigning legal rules, inter alia, explicitly recognizes four genders among human beings: male, female, DSD/intersex (khunsa), and the effeminate male (mukhannath) (Haneef, 2011). The khunsa is recognized in Islam, as the Prophet Muhammad (peace be upon him), according to the Sunnah, replied in an answer to the question about how to determine the sex of a child born with two opposite sex organs, said that the determining factor in such a case was the organ from which the child urinates (as narrated by Abu Dawud, Vol. 4, p. 228) (Haneef, 2011). The Islamic scholar, Ibn Qudamah, defined khunsa as “a person with both male and female organs or with an opening in place of a sexual organ from which he urinates.”^1^

Classical jurists have divided the khunsa into two subcategories: non-problematic/discernible (khunsa ghayr musykil/wadhih) and problematic/intractable (khunsa musykil) (Mohd. Al-Bakri, 2011; Tak, 1998). This was done in order to integrate the khunsa into the social system and law. A khunsa ghayr musykil/wadhih is a person with both male and female genitals who can be assigned a specific sex and gender based on which genital organ is the more dominant of the two. For example, if this person urinates from the penis, ejaculates semen, or grows facial hair, he can be regarded as male. Yet, if this person develops breasts and menstruation, she should be regarded as female (Haneef, 2011; Mohd. Al-Bakri, 2011; Tak, 1998).^2^

By contrast, a khunsa musykil is a person who cannot easily be categorized as either male or female, i.e., this person continues to urinate from both the penis and the vagina (Haneef, 2011; Mohd. Al-Bakri, 2011; Tak, 1998).^3^ The above are the classical definitions of khunsa musykil, but it needs to be emphasized that this religious categorization stems from an understanding of anatomy predating the present understanding of embryology and modern imaging techniques. At present, we are collaborating with Islamic scholars and other medical experts in DSD to update these Islamic definitions to align with modern understanding of anatomy. Patients with 46,XX CAH and patients with 46,XY AIS are considered to be of this second category, according to the fatwa from the Fatwa Committee National Council of Islamic Religious Affairs Malaysia in 2006. With the advancement of modern medicine and its associated technologies, however, doctors are more capable of determining a DSD individual’s appropriate sex by investigating the person’s karyotype, gonadal tissue histology, and the internal reproductive organs, and do not just depend on the appearance of the external genitalia.

### Determining Sex and Gender of a Khunsa in Islam

#### Khunsa ghayr musykil/wadhih (Non-problematic/Discernible)

Before the advent of modern medicine and the associated technologies, to determine the sex of the khunsa ghayr musykil/wadhih in Islam, one was to look for signs of “maleness” or “femaleness” in their external genitalia or in the person’s other somatic characteristics (Tak, 1998). These signs can be observed during childhood. The main sign, as decreed by the Holy Prophet (pbuh), is the organ from which the khunsa urinates.^4^

#### Khunsa musykil (Problematic/Intractable)

Khunsa musykil is a person who urinates from both genitalia at the same time and is one in whom it is difficult to ascertain the correct gender because there is no dominant male or female characteristic. Again, this definition is the classical definition which predates modern understanding of anatomy; presumably, the above definition refers to atypical external genitalia appearance. In this case, one has to wait and observe for changes in puberty to ascertain the true gender of the khunsa musykil.^5^ One of the possible changes which will indicate the person’s true sex is that the person will urinate exclusively from one of the two genitalia, or most of the urine comes out from one genital as compared to the other, or the urine stream ends from one genital rather than the other.^6^

Other changes may be that the person attains menarche or develops breasts or develops a sexual attraction to men or gets pregnant, in which case she is certainly female; or the person develops a deep voice or grows facial hair or develops a sexual attraction to women, in which case he should be designated a male. Other characteristics that may determine gender, but about which the Ulama (Muslim clerics) have conflicting opinions, include the location or anatomy of the orifice from which a person ejaculates and the number of ribs that he or she has (Tak, 1998).^6^ It is believed that females will have an extra rib compared to males as Eve (called Hawa by Muslims) was created from the Prophet Adam’s rib (Tak, 1998).

According to Shariah law, in these cases of khunsa musykil, surgery may be performed if so advised by medical experts to help ascertain the khunsa’s true sex, so that the person can be designated a certain gender in order for him or her to be able to have a good life and be able to perform his or her duties as a Muslim (Mohd. Al-Bakri, 2011). Surgery here means exploratory surgery to assess the person’s gonads or internal reproductive organs.

### Islamic Jurisprudence (Fiqh)

Imām Abū Hanīfah (d.769 C.E.), a famous Islamic scholar, defined Fiqh (Islamic Jurisprudence) as “the understanding of a person’s rights and obligations (which are directly related to his actions)” (Ebrahim, 2008). The five major Schools of Islamic jurisprudence are: Hanafī, Mālikī, Shāfī i, Hanbali, and Ja’fari. The Hanafī school is the most widespread in the Muslim world (Ebrahim, 2008). Malaysia follows the Shāfī’i school of Islamic jurisprudence.

### Gender-Oriented Aspects of Islamic Jurisprudence (Al-Fiqh)

Many aspects of Islamic rituals, rights, or obligations are gender oriented according to Islamic jurisprudence (Al-Fiqh). Examples of these are the conduct of the obligatory prayers (Solah), the Aurat (the parts of the body that need to be covered under specific circumstances), marriage roles and obligations, bathing rituals for the deceased, and the portions of wealth that can be inherited. Muslims are guided in their actions, their knowledge and understanding of their rituals, and their rights and obligations by following Al-Fiqh or Islamic jurisprudence. The various schools of Islamic jurisprudence differ in their rulings on these matters in regard to the status of khunsa individuals.

#### Prayers (Solah)

There are several differences in the way Muslim men perform their prayers (also called Solah or Solat) compared to Muslim women. Men are strongly advised to perform their daily prayers in the mosques in prayer congregations, and it is compulsory for Muslim men to pray the Jumaat (Friday afternoon prayers) in a congregation in mosques within their locality. Women are not advised to perform their daily prayers in the mosque, and it is not obligatory for them to perform the Jumaat prayers at all.

During the solah jamaah, or prayer congregation, when Muslims pray together following the lead of the imam, the position of the male adult is directly behind the imam, followed by male children, then the adult females, then the female children. Another difference concerns the Aurat. For men, the Aurat is from the navel to the knees, whereas, for women, the Aurat is the whole body except the face and the hands.

According to Islamic jurisprudence, the khunsa’s obligations in regard to the daily prayer are the same as those of the adult female, i.e., it is highly recommended that the khunsa pray at home (Tak, 1998).^7^ The khunsa’s position in the prayer congregation is right in the middle, behind the adult males and the male children and in front of the female children and adult women.^8^ This is agreed upon by all the Islamic schools of jurisprudence (Mohd. Al-Bakri, 2011).

With regard to the compulsory Jumaat prayer, it is not obligatory for the khunsa to perform this prayer together with Muslim men in the mosques, unless the khunsa’s gender has been determined to be male.^9^

Concerning the khunsa’s Aurat during prayer, if it is still uncertain to which gender the khunsa belongs, the khunsa should wear female attire (i.e., be fully covered except for the face and hands); however, if the gender has been determined, the khunsa should follow that gender’s attire accordingly (Tak, 1998).^10^

In terms of being an Imam, if the khunsa is still undecided on the gender to which he or she is assigned, then the khunsa cannot be an imam for other men (i.e., cannot lead the prayers), but the khunsa can be an imam for other khunsa and for women (Tak, 1998). Mohd. Al-Bakri (2011), however, says that a khunsa may not be able to be an imam for other khunsa as well as he/she may be a woman.

### Inheritance Rights

In Islam, both men and women are entitled to inherit wealth and assets from a deceased relative. The following verse in the Quran emphasizes this: “There is a share for men and a share for women from what is left by parents and those nearest related, whether the property be small or large–a legal share”(Sûrah An-Nisâ’4:7).

However, the portion or amount inherited by men is twice the portion or amount that is due women. The reason for this is because, in Islam, a woman’s sustenance (provision of shelter, food, clothes) is required to be provided for by men (Ahmad, 2010). A Muslim woman is not required and not responsible to provide sustenance to, or for, anyone (Ahmad, 2010). It is mandatory for a Muslim man, however, to provide sustenance to his wife and family members. Hence, a Muslim man has greater need for wealth compared to a Muslim woman, in order for him to be able to fulfill his responsibilities.

The persons entitled to the wealth and assets of a deceased Muslim include not only his or her children, parents, and spouses, but also the extended family members (Al-Khim, Al-Bugho, & Asy-Syarbaji., 2009). However, the presence of a son in the family will preclude distribution of the inheritance to other members in the family; for example, the presence of a son prevents the uncles, aunts, and grandparents from the inheritance altogether (Al-Khim et al., 2009; Dessouky, 2001).

Before Islam, the Arab community would determine the sex and gender of a khunsa based on the genitalia from which urine came out and, if the urine came out of both, the genitalia through which urine came out first.^11^ This criterion was accepted by Islam and decreed by the Holy Prophet Muhammad (pbuh).^12^ This was necessary to determine the portion of inheritance for the khunsa. Once the sex and gender of the khunsa have been determined, then the portion of inheritance is given accordingly. This is agreed upon by all of the Islamic religious scholars such as Ali, Muawiyah, Said b. Musayyab, and Jabir b. Said.^13^

In cases where the sex and gender remain undetermined, the various schools of Islamic jurisprudence as well as the various Islamic scholars differ in their opinions about the portion of inheritance that a khunsa musykil is entitled to.

The opinions of the Maliki and Hanbali schools as well as the scholars Abu Yusuf, Muhammad, Ibn Abbas, al-Sya’bi, Ibn Abi Laila, and al-Thauri are that the khunsa musykil is entitled to half of a man’s share and half of a woman’s share, because the actual gender is uncertain (Mohd. Al-Bakri, 2011). Abu Hanifah, however, has stated that the khunsa should receive the lowest and smallest portion of the inheritance (Mohd. Al-Bakri, 2011).

According to the Shāfī’i school, as the khunsa musykil is prohibited from marriage and does not bear children, such a khunsa does not have the status of father, mother, spouse, or grandparent when it comes to inheritance, but may have the status of child, sibling, and sibling of the parent of the deceased (Al-Khim et al., 2009). Once the khunsa has had children, then the sex has been determined. If the khunsa has impregnated a woman, then he is male. If the khunsa has become pregnant, then she is female. So, in either case, the person is no longer khunsa musykil but khunsa wadhih, and he or she will inherit according to the determined sex and gender. The opinion of this school of Islamic jurisprudence is that in situations where the inheritance portion is the same amount regardless of gender, then that amount should be given to the khunsa. However, in cases where the portion of inheritance depends on gender, the khunsa cannot receive any inheritance until the gender has been determined or the khunsa may receive whatever portion the other heirs and the khunsa have decided to give according to the consensus of opinions among them (Al-Khim et al., 2009).

### Bathing Rituals for the Deceased

In Islam, bathing rituals of the body of the deceased are performed in a certain way before they are prepared for burial (Kamus & Abd. Hamid, 2009). For Muslim men, the persons who are permitted to perform this bathing ritual onto the deceased comprise all other men, his wife, and his Mahram (family members whom he is forbidden to marry, such as his daughter, sister, mother, etc.). For Muslim women, the persons who are permitted to perform this bathing ritual onto the deceased comprise all other women, her husband, and her Mahram (family members whom she is forbidden to marry). There are also specific prayers which are recited for Muslim men, women, male children, and female children (Kamus & Abd. Hamid, 2009).

The various Islamic schools of jurisprudence differ in their opinions about the obligation to perform the bathing rituals, about how to bathe the deceased khunsa, and about who should perform the bathing rituals for the khunsa (Tak, 1998).

One opinion states that it is not obligatory to perform the bathing rituals for the khunsa at all; another is that it is obligatory to do so and that this responsibility falls upon the Muslim men and women in that area as well as upon the relatives of the khunsa (Tak, 1998).^14^ These opinions apply to the khunsa musykil.

According to the school of Maliki, the khunsa should be bathed properly according to the rituals, and the cleaning of the body of the deceased should not be limited to tayammum, if there are the capacity and facilities to do so. Tayammum refers to the alternative to ablution when there is no water to be found or for some reason the person performing this ritual is unable to tolerate water or water is limited. Tayammum involves using clean dust to clean one’s hands and face and is used as an alternative to ablution prior to performing prayers. According to the school of Hanafi, the khunsa cannot be bathed by either Muslim men or women, but tayammum is to be performed by the khunsa’s family members (Mohd. Al-Bakri, 2011; Tak, 1998).^15^

According to the Shāfī’i school, for a khunsa musykil who dies without having any close family members, if the khunsa is still a child, then the body may be bathed by either men or women. Conversely, if the deceased khunsa was an adult, then tayammum should be performed; yet, there is another opinion that the bathing ritual can be performed with the body covered with cloth (Mohd. Al-Bakri, 2011; Tak, 1998).

The Hanbali school is of the opinion that if the deceased khunsa is 7 years old or older and there are facilities and the capacity to do so, then the body should be bathed; however, if not, then tayammum should be performed without actually touching the body, i.e., by having the body covered with cloth (Mohd. Al-Bakri, 2011; Tak, 1998).

### The Islamic Perspective on Sex Change Surgery and Fatwas

Fatwa is defined as a formal legal opinion given by an expert in Islamic law (Masud, 1995). There are fatwas from different Islamic countries which give rulings regarding sex change surgery or gender reconstruction surgery with regard to both the khunsa and the mukhannath (the transsexual). These fatwas generally agree that gender reconstruction surgery for the khunsa is permissible in Islam but prohibited in the case of the mukhannath. For details on the position of Islamic jurists in the Sunni and the Shi’ah communities regarding the mukhannath, see Haneef (2011).

#### Fatwa From Saudi Arabia

Al Jurayyan (2011), a Professor of Paediatrics from King Saud University, Saudi Arabia, presented a set of guidelines or recommendations on this issue based on the current Islamic fatwas put forward by the senior ulama council in Saudi Arabia and the experiences of medical practitioners in Saudi Arabia (Abdullah et al., 1991; Al Herbish et al., 1996; Al Jurayyan, 2011; Couch, 1987). These fatwas are translated as follows:

1. A sex change operation [in a non-DSD individual] is totally prohibited and considered to be criminal in accordance with the Holy Quran and the Prophet’s sayings.
2. Those who have both male and female organs require further investigation and, if the evidence is more suggestive of a male gender, then it is permissible to treat the individual medically (i.e., with hormones or surgery) in order to eliminate the ambiguity and to raise him as a male and vice versa.
3. Physicians are required to explain to the child’s guardians the results of the medical investigations and whether the evidence indicates that the child is male or female in order to keep the guardians well informed.

Al Jurayyan (2011) stated that the dominant role of the male gender in the Muslim community should not overrule Islamic laws, and he emphasized that these laws should not be ignored and be given due consideration.

#### Fatwa From Malaysia

There have been several fatwas produced by the Fatwa Committee of the National Council of Islamic Religious Affairs Malaysia regarding the permissibility of genital reconstruction surgery in patients with DSD.^16^ The most recent one from November 2006 is formulated as follows:

1. For those with 46,XX CAH reared male, gender reassignment surgery to get back to the previous gender that is female is permitted in Islam because it can be treated by hormone treatment and surgery.
2. For those with 46,XY AIS reared female, getting back to the male gender through surgery or hormone treatment is quite difficult. If the patient intends to undergo surgery, it is permitted, provided that the surgery does not harm the patient psychologically or biologically.
3. For those with 46,XY AIS reared female, but diagnosed only after the person has already grown up, the person can continue a normal life and the gender is recognized from his/her [body build] and the [appearance] of the genitalia. Surgery to remove the testes (if any) is permissible to prevent the risk of cancer. The marriage of a man with a female spouse who suffers from 46,XY AIS does not need to be dissolved.
4. Medical specialists should provide explanation and advice to Muslim individuals who are affected by CAH and AIS and their parents to undergo treatment in a way that avoids any difficulties with religious regulations.

#### Fatwa From Egypt

As Dessouky (2001), a pediatric surgeon from Egypt, states, “All juristic religious opinions (fatwas) concerning the change of sex in a totally feminine or masculine human being with no physical abnormalities in his body (only due to the refusal of the person to accept his natal sex, i.e., in a transsexual) state that it is a religious doctrinal crime, as it changes ‘what God has created’.” He continues that these fatwas decreed that if both masculine and feminine characters are detected in a person (such as in a person with a DSD), the doctors should determine which characteristics are dominant and remove any other characteristic that may cause “suspicion” to achieve the best outcome for the person. Dessouky points out additional important issues in the management of Muslim patients with DSDs that still require decisions from the religious authorities, including the following:

1. which characteristic, i.e., chromosomes, gonads, phenotype, or appearance and function of the external genitalia, is the best criterion to determine whether a person is male or female;
2. the legality of performing gonadectomies or hysterectomies in patients with partial AIS and wrongly assigned males with 46,XX CAH, especially after late diagnoses.

He goes on to emphasize that, given the strength of attachment of the majority of the population to their religion, the decision on the assignment to a particular sex by both patients with DSD as well as their parents is very “precise”: no “in betweens” are tolerated; “transsexuals” are rejected by the community; and these issues are neither commonly nor freely discussed.

The sexual orientation of a khunsa can also contribute to the determination of gender. “Once a physical hermaphrodite’s dominant sexual orientation becomes clear, the hermaphrodite is thereafter considered to be of that gender. If the attraction is to males, the situation is comparable to that of a hermaphrodite whose gender has been determined as female, i.e., the person becomes a woman and should be treated like a woman. And Allah knows best” (Al-Oadah of Saudi Arabia, 2010).

## Discussion

In Malaysia, the implications of Islamic regulations for the three cases described above are as follows. In the first case, once AA has gone through the due process and received approval by the religious authorities in Malaysia for reassignment to the male gender, AA is to be regarded as male and no longer khunsa musykil. He will need to abide by the laws of Islam as a Muslim male and will be entitled to the rights of a Muslim man. He will be allowed to marry and is responsible for the sustenance and well-being of his wife. He can pray as a man, his Aurat is that of a man, and he is entitled to double the inheritance of his female siblings. BB at present is still a khunsa musykil, needs to be informed of this status, and treated accordingly, until the choice of gender has been made. CC’s gender identity is female. Thus, this person can be regarded a Muslim female and be accorded the obligations and rights as such. In all three cases, the surgeries performed were in accordance with the fatwas issued in Malaysia at that time.

In light of the discussions above, Muslim patients with DSDs and their families need to be counseled on both the societal and the religious implications of gender assignment. The recent fatwa from Malaysia has declared that gender-affirming surgery in specific DSD cases of CAH and AIS is permissible, and the fatwas from other countries also state the permissibility of surgical treatment for such patients.

However, the timing and extent of surgery should be decided upon by the individual, the parents, and the medical experts involved. Gonadectomy, according to the fatwa from Malaysia, is also permissible for the prevention of malignancy. Sexual intercourse in Islam is only allowed within the sanctity of marriage, and a person with DSD is allowed to marry, once the gender has been conclusively determined. Ethical considerations require that the potential spouse be informed of the person’s DSD condition.

Where fertility issues are concerned, assisted conception is allowed. However, all assisted reproductive manipulations that involve a third party, in the form of egg or sperm donation or surrogacy, are strictly prohibited (Ebrahim, 2008). This is because, in Islam, great importance is given to the *nasab* or the biological lineage of the child, who has to fulfill obligations toward his or her parents; in Islam, every child has a mother, and the mother is the one who carries him or her in her womb (Ebrahim, 2008). This also has implications for the inheritance laws, where children of a married man or woman have the right to portions of inherited wealth or assets.

In managing Muslim patients with DSDs, it is important not to focus purely on the medical and psychological aspects, but also to recognize the religious aspects in communities where religion plays a large part in the daily lives of the individual and the family (Al Jurayyan, 2011; Dessouky, 2001; Warne & Raza, 2008): “The clinician’s role is not to superimpose her/his cultural values on those of others, but to come to a decision that likely minimizes potential harm to the patient in his/her cultural environment” (Meyer-Bahlburg, 2001). The Muslim DSD patient may be living in a community where the Muslim culture is not dominant in which case the Islamic aspects of gender-related issues may not be recognized or considered unless the patient his/herself or the family or the clinician are aware of these and bring it up for consideration.

Thus, it makes sense to include a religious authority in the multidisciplinary team that manages these patients, as many decisions made in the course of the clinical management of families of individuals with a DSD affect the religious aspects of life and, therefore, the outcome of the individual patient, their families, and the community. It goes without saying that both the religious authorities and medical experts need to cooperate with and educate each other about the various aspects of care of the patient with a DSD. The confidentiality of information exchanged with regard to the patients and their families must be stressed, keeping in mind that the aim is the achievement of optimal outcome for the patient and families with DSD.

In conclusion, to ensure holistic care of Muslim individuals with DSD and their families, the physicians and other care providers involved in their management need to be aware of the religious aspects of gender-related issues in Islamic jurisprudence and involve religious authorities in the counseling of these patients and their families.

## Footnotes

1 Ibn Qudamah, al-Mughni, vol. 6, 221; see also Muhammad Arafah ibn Abd al-Baqi al-Dasuqi, Hashiyat al-Dasuqi ‘ala Shark Kabir (Egypt: Ihya al-Kutub al-Arabi, n.d.), vol. 4, 489.

2 For details, see al-Sarakhsi, al-Mabsut, 30, 130; Muhammad ibn Muhammad al-Hattab, Mahawib al-Jalil (Beirut: Dar al-Fikr, 1978) 6, 430; Mansur ibn Yunus al-Bahuti, Kahshshaf al—Qina’ (Beirut: Dar al-Fikr, 1982), 2, 594; al-Shirbini, Mughni al-Muhtaj, 3, 29.

3 Ibid, 4:287; al-Sarakhsi, al-Mabsut, 1:111; Hashiyat al-Dasuqi, 1:307; Ali Ibn Muhammad ibn Hazm, al-Mahalla (Beirut: Dar al-Fikr, n.d), 4:212; Ali ibn Sulayman al-Mardawi, al-insaf (Beirut: Dar Ihya al-Turath al-Arabi, 1957), 2:252.

4 Abd.Allah Ibn ‘Abd. Rahman, al-Darimi *Sunan al-Darimi*, Dar Ihya’ as-Sunnah an-Nabawiyyah, t.t., Vol 2, p. 365.

5 Ahmad, al-Husari *al-Tarikat wa al-Wasaya fi al-Fiqh al-Islami*, p. 222.

6 Muhammad Abdullah bin Ahmad b. Muhammad, Ibn Qudamah*, al-Mughni*. Vol 6, p. 336.

7 Ibid.

8 Ibid.

9 Ibid p. 52.

10 Sulaiman b. al-Asy’ath, Abu Daud, Sunan Abi Daud, Kitab al-Solat, “Bab Iza Kanu Thalath Kaif Yaqumum.” Beirut: Dar al-Kutub al-Ilmiah, 1969. Vol 1, p. 174. Also, Muhammad b. Ahmad, al-Ramli, Nihayat al-Mukhtaj, p. 89.

11 Aurangzib, Emperor of Hindustan, al-Fatawa al-alamkiriat al-Makrufah al-Fatwa al-Hindiah, Vol 6, p. 358.

12 Ahmad b. Hussin b. Ali, al-Baihaqi, *Sunan al-Kubra, Kitab al-Faraid Bab al-Mirath*, Bayrut: Majlis Dairat al-Maarif al-Uthmaniah 1352H. First print Vol 6, p. 261.

13 Muhammad Zaid, al-ibyani, *Syarh al-Ahkam al-Syariat Fi al-Usul al-Syakhsiah*, Vol 3, p. 120.

14 Ibid.

15 Wizarat al-Auqaf, Kitab Fiqh *‘ala al-Mahazib al-Arba’ah Qism al’Ibadat*, Qahirah: Matbah Dar al-Kutub al-Misriyyah, 1028. Printed p. 402.

16
www.e-Fatwa.gov.my
